# Omega-3 Fatty Acids Attenuate Renal Myostatin Expression and Mitochondrial Alterations Under Uremic Conditions

**DOI:** 10.3390/ijms27094030

**Published:** 2026-04-30

**Authors:** Su Mi Lee, Yu In Jeong, Sumin Jung, Dong Eun Yang, Seo Hee Rha, Seong Eun Kim, Won Suk An

**Affiliations:** 1 Department of Internal Medicine, Dong-A University, Busan 49201, Republic of Korea; sumilee@dau.ac.kr (S.M.L.); jui511@naver.com (Y.I.J.); eastin@dau.ac.kr (D.E.Y.); sekim@dau.ac.kr (S.E.K.); 2 Medical Science Research Center, Dong-A University, Busan 49201, Republic of Korea; sminliz210@gmail.com; 3 Department of Pathology, Dong-A University, Busan 49315, Republic of Korea; shrha@dau.ac.kr

**Keywords:** chronic kidney disease, mitochondrial dysfunction, myostatin, omega-3 fatty acids, uremia

## Abstract

Myostatin is associated with inflammatory processes; however, its renal expression and impact on mitochondrial homeostasis during chronic kidney disease (CKD) remain poorly defined. This study investigated whether omega-3 fatty acids (FAs) modulate renal myostatin and mitochondrial integrity under uremic conditions using both in vivo and in vitro models. In rats with adenine-induced CKD, omega-3 FA supplementation attenuated the increase in renal myostatin expression. Uremia was associated with impaired mitochondrial homeostasis, evidenced by decreased peroxisome proliferator-activated receptor gamma coactivator-1 alpha levels and increased dynamin-related protein 1 levels, alongside the upregulation of mitophagy and inflammatory markers. Furthermore, mitochondrial structural damage and reduced mitochondrial DNA (mtDNA) content were observed in uremic kidneys. Omega-3 FA treatment partially reversed these alterations, restored mtDNA levels, and preserved mitochondrial cristae integrity. In vitro, HK-2 cells treated with indoxyl sulfate exhibited increases in myostatin expression and mitochondrial impairment, which were mitigated by eicosapentaenoic acid, docosahexaenoic acid, or their combination. These findings suggest that omega-3 FAs exert protective effects against uremia-induced renal injury by suppressing myostatin and preserving mitochondrial homeostasis, specifically by modulating biogenesis, dynamics, and structural integrity. Consequently, omega-3 FAs may serve as a potential therapeutic strategy with which to preserve mitochondrial homeostasis in patients with CKD.

## 1. Introduction

Chronic kidney disease (CKD) is a global health concern frequently associated with progressive muscle wasting. Myostatin, or growth differentiation factor 8, a member of the transforming growth factor-β superfamily, acts as a potent inhibitor of skeletal muscle growth, thereby inducing muscle atrophy. Although elevated systemic myostatin levels are linked to muscle wasting in CKD [1,2], recent evidence suggests that its expression extends beyond the skeletal muscle to other organs, including the heart and kidneys [3,4]. However, reports on renal myostatin expression remain contradictory. Although a previous study failed to detect myostatin in the kidneys of healthy mice [5], other models have demonstrated its ectopic induction in non-muscle tissues under pathological stress [4,6]. This discrepancy likely arises from differences in the pathological milieu, as uremic toxins may trigger cellular stress pathways absent in healthy physiological states. Therefore, investigating renal myostatin expression, specifically under uremic conditions, is warranted to clarify its localized role in the progression of CKD.

Elevated myostatin levels in patients with CKD are associated with inflammatory responses and mitochondrial abnormalities in skeletal muscles [4,7,8]. Mitochondria are essential organelles for cellular energy production, and mitochondrial homeostasis is maintained through a coordinated balance between biogenesis, dynamics (fission/fusion), and mitophagy. The disruption of these processes leads to mitochondrial impairment, which is a central factor in the onset and progression of kidney diseases [8,9]. However, the specific associations between myostatin and mitochondrial biogenesis and dynamics in the kidneys under uremic conditions remain unclear.

Omega-3 fatty acids (FAs), such as eicosapentaenoic acid (EPA) and docosahexaenoic acid (DHA), possess anti-inflammatory and renoprotective properties. A previous study demonstrated that omega-3 supplementation reduced myostatin levels and inflammatory markers in tumor-bearing mice undergoing chemotherapy [10], suggesting a role in modulating myostatin expression by suppressing inflammatory cytokines. Higher levels of omega-3 FAs are associated with a slower decline in kidney function and a lower risk of incident CKD [11]. Given these potential anti-inflammatory and muscle-preserving effects, it is plausible that omega-3 FAs may help preserve renal health by modulating the myostatin–mitochondrial axis in CKD.

To our knowledge, this study is the first to identify renal myostatin induction under uremic conditions and its link to mitochondrial dysregulation. Furthermore, the modulation of this myostatin–mitochondrial axis by omega-3 FAs was investigated. In the present study, the effects of omega-3 FAs supplementation on renal myostatin expression and mitochondrial homeostasis were evaluated in both adenine-induced uremic rats and indoxyl sulfate (IS)-exposed human renal proximal tubular epithelial (HK-2) cells.

## 2. Results

### 2.1. Body Weight and Renal Function Data

Compared with the normal control group, blood urea nitrogen (BUN) and serum creatinine levels were significantly elevated in the adenine control group at both 3 and 5 weeks. Supplementation with omega-3 FAs for 2 weeks resulted in a substantial numerical reduction in BUN levels compared with that in the 5-week adenine control group. Serum creatinine levels also showed a trend toward partial attenuation following omega-3 FA treatment. No significant differences in body weight were observed among the experimental groups (Table 1).

Consistent with the biochemical data, histological analysis of kidney tissues revealed a substantial increase in the tubulointerstitial fibrosis in the adenine control group (Table 1 and Figure S1). Omega-3 FA supplementation resulted in a numerical reudction in the fibrotic area, exhibiting a marginal trend toward statistical significance (*p* = 0.06).

### 2.2. Changes in Myostatin Expression in Kidneys and HK-2 Cells

**Figure 1:**
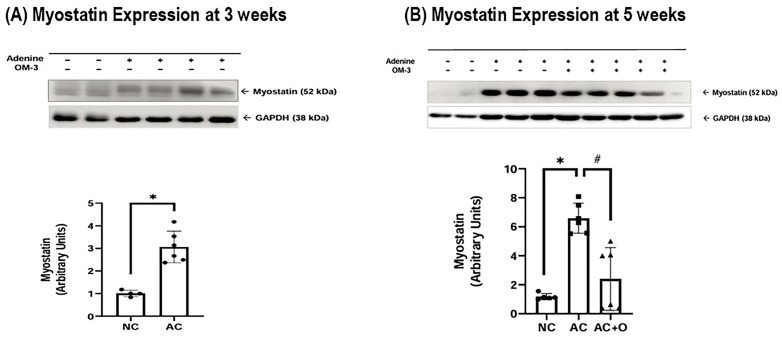
Myostatin expression in the kidneys at (**A**) 3 and (**B**) 5 weeks. Omega-3 supplementation significantly attenuated the adenine-induced increase in myostatin expression at 5 weeks. * *p* < 0.05 (mean value is significantly different from the normal control). ^#^ *p* < 0.05 (mean value is significantly different from the adenine control). OM-3, omega-3; GAPDH, glyceraldehyde 3-phosphate dehydrogenase; NC, normal control; AC, adenine control; AC + O, adenine control + omega-3 fatty acid.

**Figure 2:**
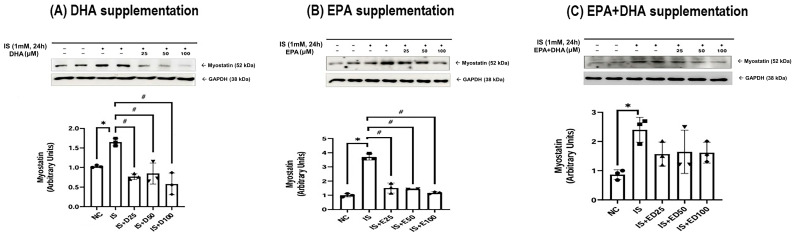
Myostatin expression in the HK-2 cells: (**A**) DHA supplementation, (**B**) EPA supplementation, and (**C**) EPA + DHA supplementation. * *p* < 0.05 (mean value is significantly different from that of the normal control). ^#^ *p* < 0.05 (mean value is significantly different from that of the adenine control). HK-2, human renal proximal tubular epithelial cells; DHA, docosahexaenoic acid; EPA, eicosapentaenoic acid; IS, indoxyl sulfate; NC, normal control; D25, DHA 25 μM; D50, DHA 50 μM; D100, DHA 100 μM; E25, EPA 25 μM; E50, EPA 50 μM; E100, EPA 100 μM; ED25, EPA + DHA 25 μM; ED50, EPA + DHA 50 μM; ED100, EPA + DHA 100 μM.

Renal myostatin expression was markedly upregulated in adenine-induced uremic rats at both 3 and 5 weeks compared to normal controls. However, this upregulation was significantly suppressed by omega-3 FAs (Figure 1). In parallel with the in vivo findings, IS exposure significantly increased myostatin expression in HK-2 cells (Figure 2). Supplementation with DHA or EPA significantly reduced IS-induced myostatin expression (Figure 2A,B).

### 2.3. Changes in Mediators Related to Mitochondrial Biogenesis and Dynamics

**Figure 3:**
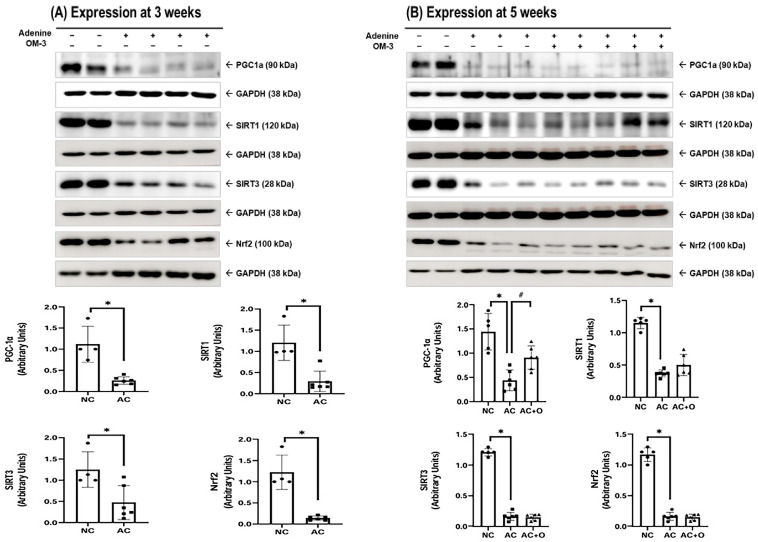
PGC-1α, SIRT1, SIRT3, and Nrf2 expression in the kidneys at (**A**) 3 and (**B**) 5 weeks. * *p* < 0.05 (mean value is significantly different from that of the normal control). ^#^ *p* < 0.05 (mean value is significantly different from that of the adenine control). OM-3, omega-3; GAPDH, glyceraldehyde 3-phosphate dehydrogenase; PGC-1α, peroxisome proliferator-activated receptor gamma coactivator-1 alpha; GAPDH, glyceraldehyde 3-phosphate dehydrogenase; SIRT1, sirtuin 1; SIRT3, sirtuin 3, Nrf2, nuclear factor erythroid-2-related factor 2; NC, normal control; AC, adenine control; AC + O, adenine control + omega-3 fatty acid.

**Figure 4:**
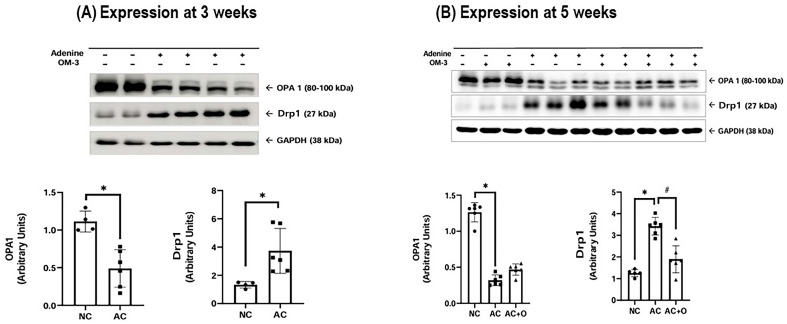
Drp1 and OPA1 expression in the kidneys (**A**) at 3 weeks and (**B**) at 5 weeks. * *p* < 0.05 (mean value is significantly different from the normal control). ^#^ *p* < 0.05 (mean value is significantly different from the adenine control). OM-3, omega-3; OPA1, optic atrophy 1; Drp1, dynamin-related protein 1; GAPDH, glyceraldehyde 3-phosphate dehydrogenase; NC, normal control; AC, adenine control; AC + O, adenine control + omega-3 fatty acid.

**Figure 5:**
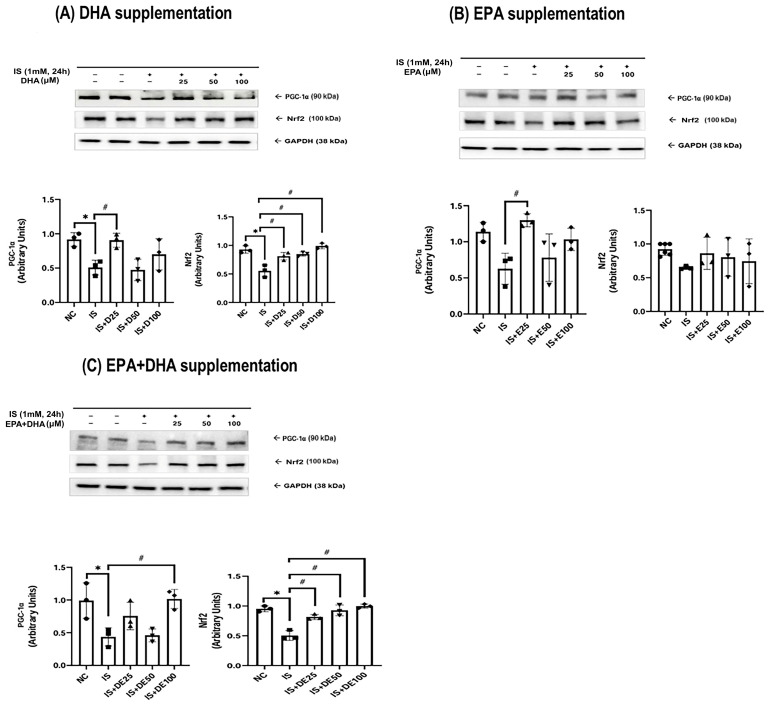
PGC-1α and Nrf2 expression in the HK-2 cells: (**A**) DHA supplementation, (**B**) EPA supplementation, and (**C**) EPA + DHA supplementation. * *p* < 0.05 (the mean value is significantly different from that of the normal control). ^#^ *p* < 0.05 (the mean value is significantly different from that of the adenine control). DHA, docosahexaenoic acid; EPA, eicosapentaenoic acid; PGC-1α, peroxisome proliferator-activated receptor gamma coactivator-1 alpha; Nrf2, nuclear factor erythroid-2-related factor 2; GAPDH, glyceraldehyde 3-phosphate dehydrogenase; NC, normal control; IS, indoxyl sulfate; D25, DHA 25 μM; D50, DHA 50 μM; D100, DHA 100 μM; E25, EPA 25 μM; E50, EPA 50 μM; E100, EPA 100 μM; ED25, EPA + DHA 25 μM; ED50, EPA + DHA 50 μM; ED100, EPA + DHA 100 μM; HK-2, human renal proximal tubular epithelial cells.

**Figure 6:**
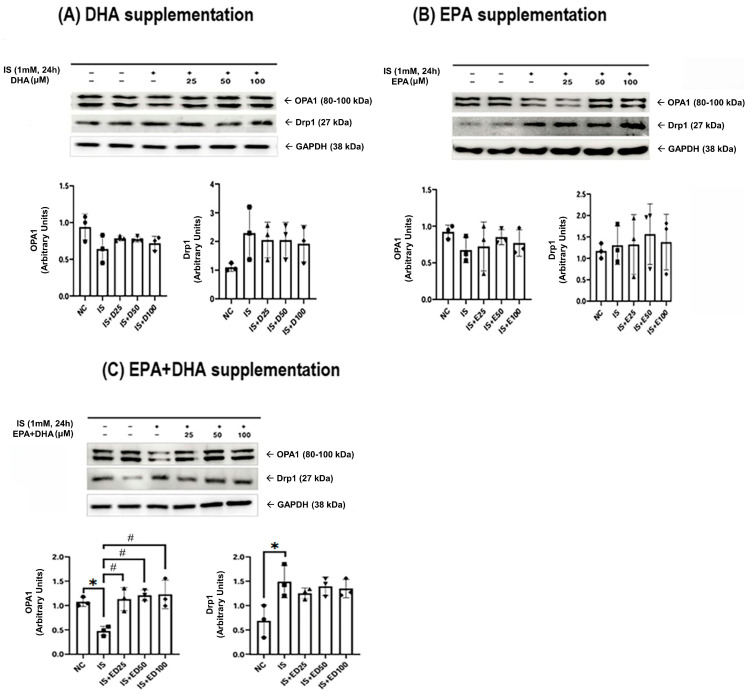
Drp1 and OPA1 in the HK-2 cells: (**A**) DHA supplementation, (**B**) EPA supplementation, and (**C**) EPA + DHA supplementation. * *p* < 0.05 (mean value is significantly different from the normal control). ^#^ *p* < 0.05 (mean value is significantly different from the adenine control). DHA, docosahexaenoic acid; EPA, eicosapentaenoic acid; IS, indoxyl sulfate; OPA, optic atrophy 1; Drp1, dynamin-related protein 1; GAPDH, glyceraldehyde 3-phosphate dehydrogenase; NC, normal control; D25, DHA 25 μM; D50, DHA 50 μM; D100, DHA 100 μM; E25, EPA 25 μM; E50, EPA 50 μM; E100, EPA 100 μM; ED25, EPA + DHA 25 μM; ED50, EPA + DHA 50 μM; ED100, EPA + DHA 100 μM.

The adenine control group exhibited a significant reduction in mitochondrial biogenesis markers, including peroxisome proliferator-activated receptor gamma coactivator-1 alpha (PGC-1α), sirtuin 1/3 (SIRT1/3), nuclear factor erythroid-2 related factor 2 (Nrf2), and fusion marker such as optic atrophy 1 (OPA1). Conversely, the fission protein dynamin-related protein 1 (Drp1) was upregulated at both 3 and 5 weeks (Figure 3 and Figure 4). Omega-3 FA supplementation specifically restored PGC-1α and Drp1 levels in the kidneys. Similarly, in HK-2 cells, IS-induced downregulation of PGC-1α, Nrf2, and OPA1, concurrent with Drp1 upregulation, was observed (Figure 5 and Figure 6). DHA or the combination treatment significantly restored Nrf2 and OPA1 expression.

### 2.4. Changes in Mediators Related to Inflammation and Mitophagy

**Figure 7:**
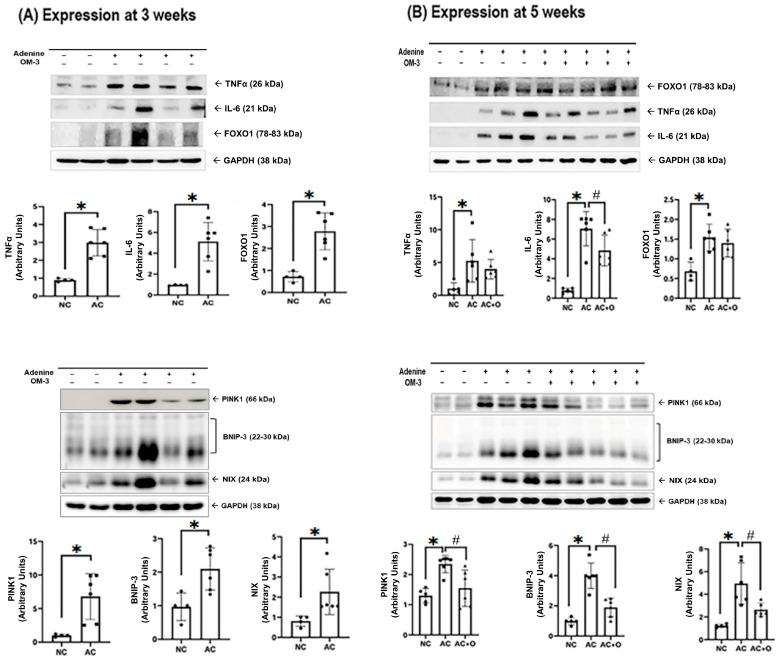
TNFα, IL-6, FOXO1, PINK1, BNIP3, and NIX expression in the kidneys at (**A**) 3 and (**B**) 5 weeks. * *p* < 0.05 (the mean value is significantly different from that of the normal control). ^#^ *p* < 0.05 (the mean value is significantly different from that of the adenine control). OM-3, omega-3; TNF-α, tumor necrosis factor-alpha; IL-6, interleukin-6; FOXO1, forkhead box O1; GAPDH, glyceraldehyde 3-phosphate dehydrogenase; NC, normal control; AC, adenine control; AC + O, adenine control + omega-3 fatty acid; PINK1, phosphatase and tensin homolog-induced putative kinase 1; BNIP3, Bcl-2/adenovirus E1B 19 kDa-interacting protein-3; NIX, Nip3-like protein X.

Uremic conditions induced a robust increase in inflammatory cytokines such as tumor necrosis factor-alpha (TNF-α) and interleukin-6 (IL-6), transcription factors such as forkhead box O1 (FOXO1), and mitophagy-related markers such as phosphatase and tensin homolog-induced putative kinase 1 (PINK1), bcl-2/adenovirus E1B 19 kDa interacting protein-3 (BNIP3), and Nip3-like protein X (NIX) in the kidneys. Notably, the levels of IL-6 and mitophagy-related markers were significantly restored after 2 weeks of omega-3 FA supplementation (Figure 7).

### 2.5. Changes in Mitochondrial DNA Contents and Cell Viability in HK-2 Cells

**Figure 8:**
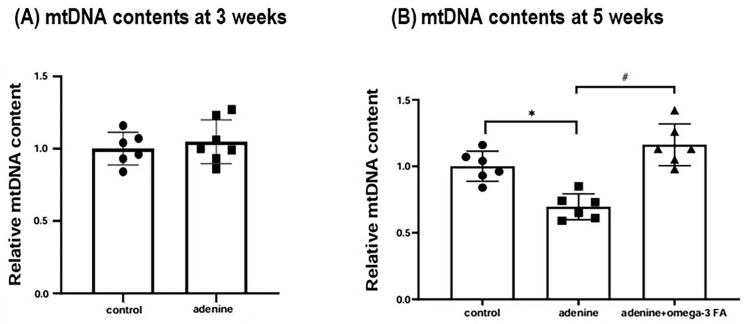
mtDNA contents in the kidneys at (**A**) 3 and (**B**) 5 weeks. * *p* < 0.05 (mean value is significantly different from the normal control). ^#^ *p* < 0.05 (mean value is significantly different from the adenine control). mtDNA, mitochondrial DNA; FA, fatty acid.

**Figure 9:**
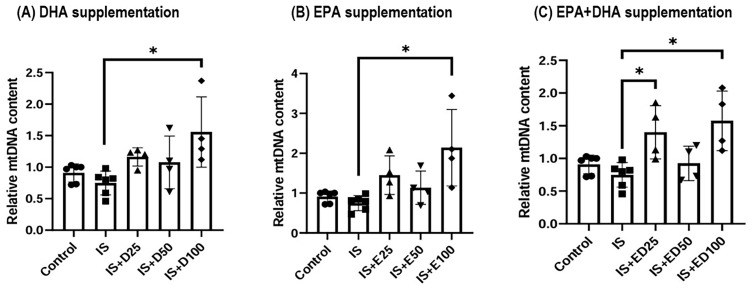
mtDNA contents in the HK-2 cells: (**A**) DHA supplementation, (**B**) EPA supplementation, and (**C**) EPA + DHA supplementation. * *p* < 0.05 (mean value is significantly different from the normal control). mtDNA, mitochondrial DNA; DHA, docosahexaenoic acid; EPA, eicosapentaenoic acid; IS, indoxyl sulfate; D25, DHA 25 µM; D50, DHA 50 µM; D100, DHA 100 µM; E25, EPA 25 µM; E50, EPA 50 µM; E100, EPA 100 µM; ED25, EPA + DHA 25 µM; ED50, EPA + DHA 50 µM; ED100, EPA + DHA 100 µM.

**Figure 10:**
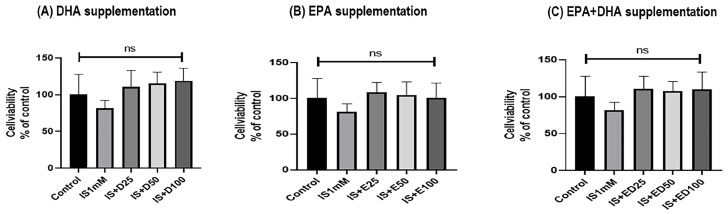
Cell viability in HK-2 cells: (**A**) DHA supplementation, (**B**) EPA supplementation, and (**C**) EPA + DHA supplementation. HK-2, human renal proximal tubular epithelial cells; DHA, docosahexaenoic acid; EPA, eicosapentaenoic acid; IS, indoxyl sulfate; D25, DHA 25 µM; D50, DHA 50 µM; D100, DHA 100 µM; E25, EPA 25 µM; E50, EPA 50 µM; E100, EPA 100 µM; ED25, EPA + DHA 25 µM; ED50, EPA + DHA 50 µM; ED100, EPA + DHA 100 µM; ns, not significant.

Renal mitochondrial DNA (mtDNA) content was significantly depleted at 5 weeks in uremic rats but was effectively restored by omega-3 FAs (Figure 8). In HK-2 cells, although IS exposure did not significantly alter mtDNA content; high-dose supplementation with DHA, EPA, or a combination treatment significantly increased mtDNA levels (Figure 9). Notably, cell viability remained unaffected by IS or omega-3 FA treatment (Figure 10).

### 2.6. Changes in Transmission Electron Microscopy Findings

**Figure 11:**
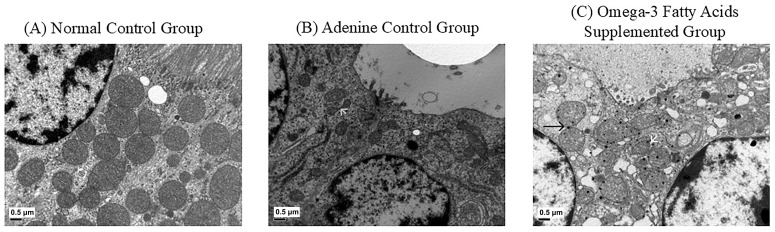
Morphological changes in mitochondria at 5 weeks in the renal tubular cells. (**A**) Normal control group showing well-preserved mitochondria with intact cristae. (**B**) Adenine control group displaying mitochondrial swelling, vacuolation, and fragmented cristae. The white arrow indicates an event of mitochondrial fission. (**C**) Omega-3 fatty acid (FA)-supplemented group showing partially attenuated mitochondrial alterations. The black arrow indicates mitochondrial fusion, whereas the white arrow represents fragmented mitochondria. Scale bar = 0.5 μm.

Figure 11 illustrates the ultrastructural changes in mitochondrial morphology. The control group exhibited well-preserved tubular cells with organized cristae (Figure 11A). In contast, the adenine control group showed mitochondrial swelling, vacuolation, and fragmented cristae (Figure 11B). In the omega-3 FA group, mitochondrial alterations were partially attenuated, showing qualitative improvements in the cristae structure (Figure 11C). These morphological observations align with the improved mtDNA/nuclear DNA (nDNA) ratio observed in both uremic rats and HK-2 cells (Figure 8 and Figure 9).

## 3. Discussion

In this study, renal myostatin induction was identified under uremic conditions, both in vivo and in vitro. In the adenine-induced CKD model, renal myostatin was significantly upregulated at both 3 and 5 weeks. Similarly, IS treatment induced myostatin expression in HK-2 cells. Although myostatin is primarily recognized as a muscle-derived factor associated with inflammatory processes in CKD, recent evidence suggests that it is also locally expressed in the kidneys. In human diabetic nephropathy, myostatin mRNA and protein levels are markedly elevated in the glomerular and tubulointerstitial compartments, where their expression correlates with interstitial fibrosis [4]. These findings reinforce the notion that uremic toxins directly stimulate renal myostatin, supporting a role for local myostatin as a key regulator of renal homeostasis.

Omega-3 FAs possess established anti-inflammatory and renoprotective properties, primarily through the modulation of inflammatory pathways [12]. Clinical evidence has indicated that higher levels of seafood-derived omega-3 FAs are associated with a lower risk of incident CKD and a slower decline in kidney function [11]. In addition to renal protection, omega-3 FAs exert beneficial effects on muscle health. A previous study reported that 6-month supplementation with omega-3 FA in healthy older adults significantly improved muscle mass and performance [13]. These observations suggest that omega-3 FAs attenuate myostatin expression and activity, thereby promoting protein synthesis and reducing inflammation [14]. Consistent with this, the present study demonstrated that omega-3 FA supplementation significantly decreased renal myostatin expression and levels of inflammatory markers such as IL-6, attenuating the decline in renal function in vivo.

Mitochondrial homeostasis, maintained by the balance among biogenesis, dynamics, and mitophagy, is essential for cellular function [15]. PGC-1α, SIRT1, SIRT3, and Nrf2 are key regulators of mitochondrial biogenesis and antioxidant defense [16]. Disruption of these processes leads to mitochondrial impairment, which plays a central role in the onset and progression of cardiorenal diseases [17,18,19]. In the current study, adenine-induced uremia significantly downregulated these markers at both 3 and 5 weeks in vivo, indicating severe impairment of mitochondrial quality control. Notably, omega-3 FA supplementation partially restored PGC-1α expression in vivo, whereas the Nrf2 response was more pronounced in vitro.

This discrepancy between in vivo and in vitro responses, particularly regarding Nrf2, may be attributed to several factors. First, the systemic uremic environment in vivo is more complex than a controlled in vitro setting, as animal models are exposed to a broader spectrum of uremic toxins and chronic inflammatory cytokines beyond IS, suppressing the Nrf2/SIRT signaling axis to a degree that a 2-week treatment cannot fully reverse. Second, differences in omega-3 FAs’ bioavailability and tissue distribution may contribute further. HK-2 cells are directly exposed to controlled concentrations of DHA/EPA, whereas in vivo, these FAs undergo systemic metabolism, potentially leading to lower effective concentrations in renal tissues. Finally, the progressive nature of adenine-induced renal injury may require a more prolonged intervention to observe significant recovery in broader biogenesis markers, whereas acute in vitro IS exposure allows for more rapid observation of the protective effects of omega-3-mediated protection.

Uremia also affects mitochondrial dynamics and quality control. Excessive mitochondrial fission mediated by Drp1 leads to mitochondrial fragmentation and injury [20,21]. Our results show that uremia increased Drp1 expression, which was reversed by omega-3 FA treatment. Interestingly, although omega-3 FA restored mtDNA levels at 5 weeks, no significant decrease was observed at 3 weeks or in the in vitro IS model. This result suggests that the reduction in mtDNA levels may be a delayed response compared to the more immediate alterations in mitochondrial biogenesis and dynamic markers.

Mitophagy is a selective form of autophagy that removes damaged mitochondria to maintain quality control. Dysregulated or injured mitochondria generate excessive mitochondrial reactive oxygen species, which activate mitophagy [8,9]. This process is primarily regulated through the PINK1-Parkin pathway and alternative receptor-mediated pathway involving BNIP3 and NIX. In this study, uremia significantly upregulated mitophagy-related markers, including FOXO1, PINK1, BNIP3, and NIX, at 3 and 5 weeks. This upregulation suggests a compensatory response to severe mitochondrial damage that warrants confirmation with functional assays such as metabolic flux analysis to confirm the absolute metabolic shift. Notably, levels of FOXO1, a transcription factor known to enhance PINK1/Parkin-dependent mitophagy, increased alongside pro-inflammatory cytokines such as TNF-α and IL-6. Uremia-induced mitochondrial fragmentation, reflected by increased Drp1 levels, and the associated rise of mitophagy markers were significantly attenuated by omega-3 FAs, indicating that omega-3 FAs reduce mitochondrial stress, thereby alleviating the demand for sustained compensatory mitophagy.

The ultrastructural analyses further supported these molecular changes. Transmission electron microscopy (TEM) revealed severe mitochondrial structural damage in the adenine control group, characterized by swelling and cristae disruption. In contrast, the omega-3 FA-supplemented group exhibited significantly improved mitochondrial architecture. Attenuation of IL-6, FOXO1, and mitophagy markers following omega-3 FA treatment indicates that omega-3 FAs mitigate CKD-related mitochondrial injury and inflammation. Altogether, these findings suggest that omega-3 FAs suppress renal myostatin expression and preserve mitochondrial integrity by modulating biogenesis, dynamics, and quality control pathways under uremic conditions.

This study has some limitations. First, the lack of direct gain- and loss-of-function experiments for myostatin precludes definitive causal inference. Nevertheless, considering that previous reports have identified myostatin as a potent inducer of oxidative stress and inflammation in skeletal muscle and kidney tissues [2,4], our results provide a foundational basis for the myostatin–mitochondrial axis in uremic kidneys. Second, mitochondrial assessment relied mainly on marker expression and qualitative morphology evaluation via TEM rather than direct functional assays or quantitative morphometry. Finally, the use of only male rats minimized hormonal interference but also limited the generalizability to both sexes. Future studies using myostatin siRNA or overexpression models, combined with comprehensive mitochondrial functional assays and quantitative structural analysis in both male and female models, are warranted to establish causality and validate the proposed metabolic shifts in uremic mitochondria across both sexes.

In conclusion, omega-3 FAs suppress myostatin expression and attenuate mitochondrial alterations under uremic conditions, supporting their potential as a therapeutic strategy for preserving mitochondrial homeostasis in CKD.

## 4. Materials and Methods

### 4.1. Animals and Experimental Design

To induce CKD, 8-week-old male Sprague-Dawley rats were maintained on a diet containing 0.75% adenine and 2.5% protein, supplemented with cholecalciferol 3000 IU/kg/week (Solgar, Leonia, NJ, USA) for 3 weeks. Subsequently, animals were randomly assigned by body weight and allocated into experimental groups. The treatment phase involved a 2.5% protein diet administered via gastric gavage for 2 weeks, consisting of either saline (adenine control) or omega-3 FA (300 mg/kg/day; Omacor, Kuhnil Pharm., Seoul, Republic of Korea). Each 1-g capsule of Omacor consists of 460 mg of EPA and 380 mg of DHA as ethyl esters. The dosage of 300 mg/kg/day was determined based on a previous study [22].

The experimental groups were as follows: normal control (n = 5), adenine control killed at 3 (n = 6) and 5 weeks (n = 5), and omega-3 FA group killed at 5 weeks (n = 5). All procedures received approval from the Institutional Animal Care and Use Committee of Dong-A University (DIACUC-21-39).

### 4.2. Histological Analysis and Quantification of Tubulointerstitial Fibrosis

After the rats were killed, their kidney tissues were harvested and cleansed with saline containing heparin. Specimens were fixed in periodate-lysine-paraformaldehyde and encased in paraffin blocks. Following the removal of wax, 4-μm sections underwent periodic acid-Schiff staining. To facilitate objective analysis, the stained slides were digitized using an Aperio Scan Scope (Aperio Technologies, Vista, CA, USA).

The severity of tubulointerstitial injury was assessed by measuring areas exhibiting tubular dilatation, tubular atrophy, and interstitial fibrosis using CaseViewer (version 2.4, 3DHISTECH LTd., Budapest, Hungary) and Image-Pro Plus software (version 7.0, Media Cybernetics, Rockville, MD, USA). The percentage of tubulointerstitial fibrosis was determined by dividing the combined pathological area by the total stained tissue area across five random high-power fields (20× objective).

### 4.3. Cell Culture

HK-2 cells from ATCC (Manassas, VA, USA) were maintained in RPMI 1640 medium supplemented with 10% fetal bovine serum, with the addition of 100 U/mL penicillin and 100 μg/mL streptomycin (Welgene, Gyeongsan, Republic of Korea). The cells were cultured at 37 °C in a 5% CO_2_ humidified incubator. Upon reaching 80–90% confluence, the cells were treated with DHA, EPA, or a DHA + EPA mixture (2:3 ratio) (Sigma-Aldrich, St. Louis, MO, USA) at final concentrations of 25, 50, or 100 μM. IS (Sigma-Aldrich) was simultaneously administered at a concentration of 1 mM for 24 h. To prepare the treatment, FAs were dissolved in 100% ethanol and complexed with 3 mM bovine serum albumin (BSA) in a 4:1 molar ratio (FA/BSA). The final ethanol concentration in the medium was maintained below 0.1%, and the normal control group received an equivalent concentration of the BSA-ethanol vehicle.

### 4.4. Protein Extraction and Western Blotting

Total protein was extracted using RIPA buffer containing 1% Nonidet P-40, 1 mM ethylenediaminetetraacetic acid, protease inhibitors, phosphatase inhibitors, and 1 mM phenylmethylsulfonyl fluoride. A BCA Protein Assay Kit (Thermo Fisher Scientific, Waltham, MA, USA) was used to measure the total protein concentration in each sample.

Equal amounts of protein (20–30 μg) were separated via 8–10% sodium dodecyl sulfate-polyacrylamide gel electrophoresis (SDS-PAGE) and transferred to nitrocellulose (NC) membranes. After blocking with 5% (*w*/*v*) skim milk for one hour, membranes were incubated overnight at 4 °C with primary antibodies against myostatin, PGC-1α, SIRT1, Nrf2 (sc-13032), PINK1, BNIP3, NIX, TNF-α, IL-6 and GAPDH (Santa Cruz Biotechnology, Santa Cruz, CA, USA); Drp1 and OPA1 (BD Bioscience, Franklin Lakes, NJ, USA); and SIRT3 and FOXO1 (Cell Signaling Technology, Danvers, MA, USA) [3,4].

Protein signals were detected using Super Signal West Pico Chemiluminescence Substrate (Thermo Fisher Scientific) and visualized using an AMERSHAM ImageQuant 800 system (GE Healthcare Bio-Sciences AB, Uppsala, Sweden). Protein band densities were normalized to those of GAPDH as an internal control. Signals were quantified using ImageJ software (version 1.53t, National Institutes of Health, Bethesda, MD, USA).

### 4.5. Measurement of mtDNA Content

Relative mtDNA levels were measured by quantitative real-time polymerase chain reaction (qRT-PCR). Total DNA was isolated from the tissue samples using TRIzol reagent (Thermo Fisher Scientific) according to the manufacturer’s protocol. Relative mtDNA content was determined by normalizing mtDNA to nDNA. The rat mtDNA sequence was targeted for renal tissues, whereas the human MT-ND1 gene was used for HK-2 cells. Normalization was performed using GAPDH (for rats) or RPLP0 (for humans) as internal controls. qRT-PCR was conducted using SYBR Green PCR Master Mix (Enzynomics, Daejeon, Republic of Korea) on an ABI 7500 Real-Time PCR System (Applied Biosystems, Foster City, CA, USA). The primer sequences designed using Primer3 v. 2.6.1 and mFold v. 3.6 software were as follows:

- Rat mtDNA: sense 5′-GGT TCT TAC TTC AGG GCC ATC A-3, antisense 5′- TGA TTA GAC CCG TTA CCA TCG A-3
- Rat GAPDH: sense 5′-CAA GAA GGT GAA GCA GG-3′, antisense 5′-GGT GGA AGA ATG GGA GTT GC-3′
- Human mtDNA (MT-ND1): sense 5′-CGG GCT ACT ACA ACC CTT CG-3′, antisense 5′-GCG ATG GTG AGA GCT AAG GT-3′
- Human RPLP0: sense 5′-CAG GTG TTC GAC AAT GGC AG-3′, antisense 5′-CAC TGG CAA CAT TGC GGA C-3′

### 4.6. Cell Viability Assay

Cell viability was assessed using an MTT assay (Thermo Fisher Scientific). HK-2 cells (5 × 10^4^ cells/mL) were seeded into 48-well plates and treated with varying concentrations of DHA, EPA, or DHA/EPA mixture (25, 50, or 100 μM) for 24 h. As described in Section 4.2, the FAs were complexed with 3 mM BSA as a vehicle. The untreated normal control group was incubated with an equivalent concentration of the BSA vehicle. Following treatment, 10 μL of MTT solution was added to each well, and the cells were incubated for an additional hour at 37 °C. The culture medium was then carefully removed, and the resulting formazan crystals were dissolved in 200 μL of dimethyl sulfoxide. The optical density (OD) was measured at 570 nm using a BioTek Synergy HTX microplate reader (BioTek Instruments, Winooski, VT, USA).

### 4.7. Transmission Electron Microscopy

To minimize mitochondrial damage and ensure optimal structural preservation, the rats underwent cardiac perfusion. Briefly, the thoracic cavity was surgically opened to expose the heart, and blood was flushed with phosphate-buffered saline, followed by perfusion fixation with 4% paraformaldehyde for 20 min. Kidney tissues were then harvested and fixed in 2.5% glutaraldehyde in 0.1 M cacodylate buffer (pH 7.2) at 4 °C for 2 h, followed by post-fixation in 1% osmium tetroxide at room temperature. After dehydration using a graded ethanol series, tissues were embedded in epoxy resin and sectioned into ultrathin slices (approximately 70 nm). These sections were visualized using a transmission electron microscope (Talos F200X, Thermo Fisher Scientific) operated at 200 kV in the bright-field mode to obtain high-resolution images of the renal ultrastructure.

### 4.8. Statistical Analysis

Results are expressed as the mean ± standard deviation. To evaluate statistical significance, intergroup comparisons were performed using the Kruskal–Wallis test, followed by Dunn’s multiple comparison test for post hoc analysis. Statistical comparisons were performed between the normal control and the adenine-induced CKD group to evaluate the induction of uremia. To assess the effects of omega-3 FAs, the omega-3 FA-treated groups were compared against the adenine control group. Similarly, for in vitro data, IS-treated cells were compared with untreated controls, and omega-3 FA-treated groups were compared with the IS-only group. Statistical significance was defined as *p* < 0.05. All calculations were performed using GraphPad Prism 8.0 (GraphPad Software, San Diego, CA, USA).

## Figures and Tables

**Table 1:** Characteristics of the rats in each experimental group.

	Normal Control (n = 5)	Adenine Control at 3 Weeks (n = 6)	Adenine Control at 5 Weeks (n = 5)	Adenine Control with Omega-3 Fatty Acid at 5 Weeks (n = 5)	*p* Value
Final Body Weight (g)	279.8 ± 13.5	276.8 ± 11.0	251.6 ± 29.2	281.4 ± 25.7	0.118
Blood urea nitrogen (mg/dL)	5.3 ± 3.4	65.0 ± 28.0 *	64.4 ± 46.7 *	26.7 ± 10.4 *^#^	0.009
Creatinine (mg/dL)	0.4 ± 0.1	4.1 ± 1.0 *	5.1 ± 2.0 *	3.6 ± 0.6	<0.001
Tubulointerstitial fibrosis (%)	2.6 ± 1.5	50.0 ± 7.1 *	57.0 ± 10.4 *	41.0 ± 9.6 *	0.003

Data are expressed as means ± standard deviation. The nonparametric Kruskal–Wallis test, followed by Dunn’s multiple comparison test for post-hoc analysis, was used to compare continuous variables among the groups. * *p* < 0.05 (mean values are significantly different from those of the control group). ^#^ *p* < 0.05 (mean values are significantly different from those of the adenine control group at 3 weeks).

## Data Availability

The data presented in this study are available on request from the corresponding author. The data are not publicly available due to institutional policy regarding the sharing of raw research data.

## Supplementary Materials

The following supporting information can be downloaded at https://www.mdpi.com/article/10.3390/ijms27094030/s1.

## Abbreviations

The following abbreviations are used in this manuscript:

AC	Adenine control
BCA	Bicinchoninic acid
BNIP3	Bcl-2/adenovirus E1B 19kDa-interacting protein 3
BUN	Blood urea nitrogen
CKD	Chronic kidney disease
DHA	Docosahexaenoic acid
Drp1	Dynamin-related protein 1
EPA	Eicosapentaenoic acid
FAs	Fatty acids
FBS	Fetal bovine serum
FOXO1	Forkhead box O1
GAPDH	Glyceraldehyde 3-phosphate dehydrogenase
HK-2	Human renal proximal tubular epithelial cells
IL-6	Interleukin-6
IS	Indoxyl sulfate
mtDNA	Mitochondrial DNA
MTT	3-(4,5-dimethylthiazol-2-yl)-2,5-diphenyltetrazolium bromide
NC	Normal control
nDNA	nuclear DNA
NIX	Nip3-like protein X
Nrf2	Nuclear factor erythroid-2 related factor 2
OPA1	Optic atrophy 1
PGC-1α	Peroxisome proliferator-activated receptor gamma coactivator-1 alpha
PINK1	Phosphatase and tensin homolog-induced putative kinase 1
qRT-PCR	Quantitative real-time polymerase chain reaction
SD	Standard deviation
SDS-PAGE	Sodium dodecyl sulfate-polyacrylamide gel electrophoresis
SIRT1	Sirtuin 1
SIRT3	Sirtuin 3
TEM	Transmission electron microscopy
TNF-α	Tumor necrosis factor alpha
