# Supplementary material for: Omega-3 Fatty Acids Attenuate Renal Myostatin Expression and Mitochondrial Alterations Under Uremic Conditions
Source: Int J Mol Sci. 2026 Apr 30;27(9):4030. doi: 10.3390/ijms27094030 (PMC13163538; doi:10.3390/ijms27094030)

Supplementary Figure1. Histopathological changes in the kidney tissues of adenine-induced uremic rats. Compared to the normal control (A), the adenine control group at 3 weeks (B) and 5 weeks (B) exhibited pathological features, including tubular dilatation, tubular atrophy, and interstitial fibrosis. (D) Omega-3 fatty acid supplementation group, exhibiting less severe tubulointerstitial alterations compared to the 5-week adenine control. These qualitative observations are consistent with the numerical trends presented in Table 1. Scale bar = 20 μM.

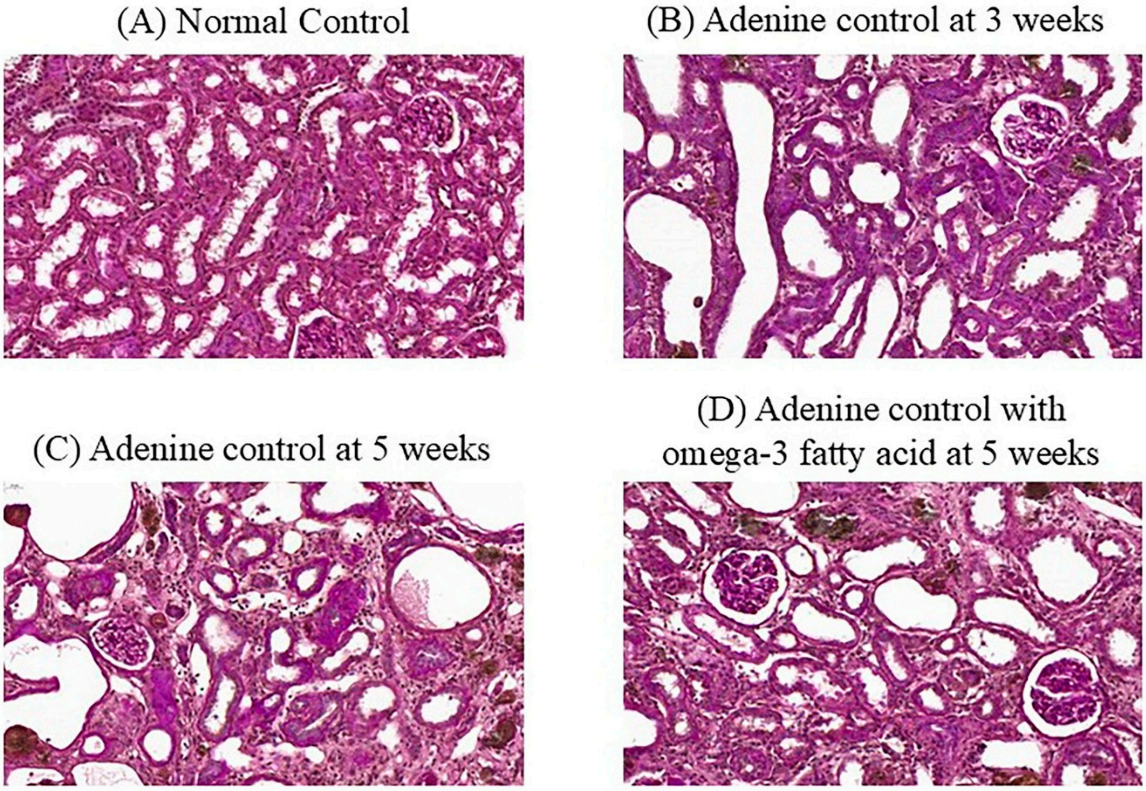

Supplement: Supplementary file 1 [file ijms-27-04030-s001.zip › ijms-4244769-supplementary.pdf]
